# Hallmarks of Cancer Cachexia: Sexual Dimorphism in Related Pathways

**DOI:** 10.3390/ijms26093952

**Published:** 2025-04-22

**Authors:** Setareh Satari, Inês N. R. Mota, Ana Carolina Leão Silva, Haissa Oliveira Brito, Paula A. Oliveira, Rui Miguel Gil da Costa, Rui Medeiros

**Affiliations:** 1 Molecular Oncology and Viral Pathology Group, Research Center of IPO Porto (CI-IPOP)/CI-IPOP@RISE (Health Research Network), Portuguese Oncology Institute of Porto (IPO Porto)/Pathology and Laboratory Medicine Dep./Clinical Pathology, Porto Comprehensive Cancer Center Raquel Seruca (Porto.CCC Raquel Seruca), 4200-072 Porto, Portugal; satarisetareh.md@gmail.com (S.S.); inesnrmota@gmail.com (I.N.R.M.); acleao.nutri@gmail.com (A.C.L.S.); rui.costa@ufma.br (R.M.G.d.C.); 2 Faculty of Medicine, University of Porto (FMUP), 4200-319 Porto, Portugal; 3 The Institute of Public Health, University of Porto (ISPUP), Rua das Taipas 135, 4050-600 Porto, Portugal; 4 Faculty of Sciences, University of Porto (FCUP), 4169-007 Porto, Portugal; 5 Research Center For Experimental and Clinical Physiology and Pharmacology (NEC)/Centre for the Research and Technology of Agro-Environmental and Biological Sciences (CITAB)/Bioanalysis Lab (LaBIO), Federal University of Maranhão (UFMA), São Luís 65080-805, Brazil; haissa.brito@ufma.br; 6 Centre for the Research and Technology of Agro-Environmental and Biological Sciences (CITAB), Inov4Agro, University of Trás-os-Montes and Alto Douro (UTAD), 5000-801 Vila Real, Portugal; pamo@utad.pt; 7 Laboratory for Process Engineering, Environment, Biotechnology and Energy (LEPABE), Faculty of Engineering, University of Porto, Rua Dr. Roberto Frias, 4200-465 Porto, Portugal; 8 ICBAS-School of Medicine and Biomedical Sciences, University of Porto, 4050-313 Porto, Portugal; 9 Biomedical Research Center, Faculty of Health Sciences of the Fernando Pessoa University, 4249-004 Porto, Portugal; 10 ECO-European Cancer Organization, 1040 Brussels, Belgium; 11 Research Department of the Portuguese League Against Cancer—Regional Nucleus of the North (Liga Portuguesa Contra o Cancro—Núcleo Regional do Norte), 4200-172 Porto, Portugal

**Keywords:** cancer, cachexia, hallmarks, sexual dimorphism, sex hormones

## Abstract

Cancer-associated cachexia (CAC), also known as wasting syndrome, is a systemic condition that affects multiple tissues and organs via a variety of metabolic pathways. Systemic inflammation, progressive weight loss, depletion of adipose tissue, and skeletal muscle impairment are some of the hallmark features of cachexia. Despite various studies on the clinical features of CAC, the complexity of the syndrome continues to pose significant challenges in clinical practice, leading to late diagnoses and the absence of a standardised treatment. Men and women respond differently to CAC, which may be prompted by the pre-existing physiologic sex differences. This review presents the sexual dimorphism associated with the hallmark pathways involved in CAC. A comprehensive understanding of sexual dimorphism in these pathways could drive research on cachexia to prioritise the inclusion of more females in related studies in order to achieve personalised sex-based therapeutic approaches and, consequently, enhance treatment efficacy and better patient outcomes.

## 1. Introduction

Cancer ranks among the top causes of mortality globally, responsible for approximately 10 million fatalities in 2022, which is nearly one out of every six deaths [1]. Men have a slightly higher lifetime risk of invasive cancer (40.9%) compared to women (39.1%), primarily due to greater exposure to occupational carcinogenic and behavioural factors like smoking. However, endogenous hormone exposure, immune function, and response also play significant roles in this sex-based difference [2].

**Figure 1:**
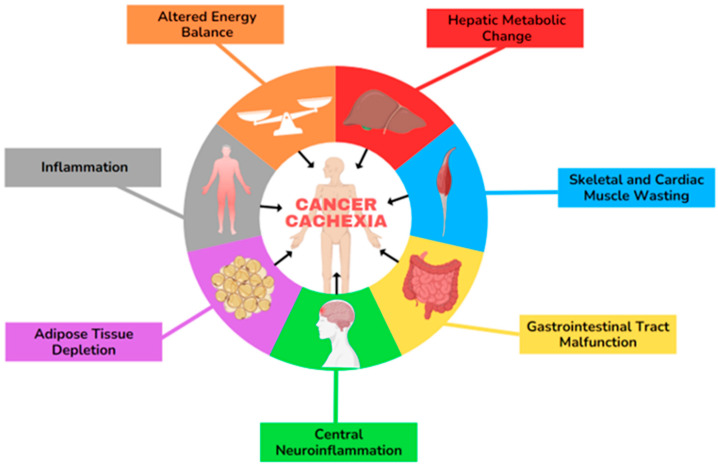
The hallmarks of cancer-associated cachexia (CAC). This illustration encompasses six hallmark features involved in the development of CAC.

Cancer-associated cachexia (CAC) is a multifactorial syndrome with poorly understood mechanisms, including systemic inflammation, loss of appetite, anorexia, and weight loss. The progression involves hallmark features of multiple pathways related to adipose tissue (AT) alterations, muscle atrophy, energy-expenditure imbalance, gastrointestinal (GI) tract malfunction, and hepatic metabolic changes, which are represented in Figure 1 [3,4,5,6,7]. Cachexia predominantly affects individuals with advanced pancreatic, lung, and bladder cancer, but it also commonly occurs in those with head and neck, colorectal, ovarian, and liver malignancies [8,9,10,11]. According to the latest ASCO guideline, cachexia is defined as more than 5% weight loss over the last 6 months or a BMI of 20 kg/m^2^ with ongoing > 2% weight loss or depletion of muscle mass and >2% weight loss. Cachexia can involve up to 80% of the patients with advanced cancer and significantly impacts individuals’ survival [12]. Cancer cachexia progresses through three clinical stages: pre-cachexia (marked by minimal weight loss (≤5% of body mass)), cachexia, and refractory cachexia, though not all patients experience each phase. The refractory cachexia stage is particularly challenging, as it is characterised by severe metabolic dysfunction, resistance to anticancer treatments, poor performance status, and a life expectancy of less than three months [6,13]. Sex differences in physiological processes and diseases primarily arise from distinctions in sex chromosomes and sex hormones [14]. Research indicates that DNA methylation and histone modifications differ between sexes, and quantitative trait locus analyses reveal sex-specific genetic regulation of traits [15,16]. Moreover, Lim et al., in their review on the development and progression of CAC, highlight that the cancer incidence is approximately 20% higher in males compared to females, with notable differences in the male-to-female incidence ratio across various primary malignant tumours, including liver, bladder, oral cavity, oesophageal, and thyroid cancers in the United States. Additionally, mortality rates for malignant cancers are nearly 30% higher in males across different age groups. Cancer-induced cachexia affects 40–50% of female patients and 40–60% of male patients over the age of 60, with severe muscle loss being significantly more prevalent in males (61%) than in females (31%) [13,17]. Klassen et al. studied palliative chemotherapy for advanced pancreatic cancer and found that both treatment type and sex differences significantly impact whether adipose or muscle loss is more pronounced. In their study, men showed more muscle mass loss than women [18].

**Figure 2:**
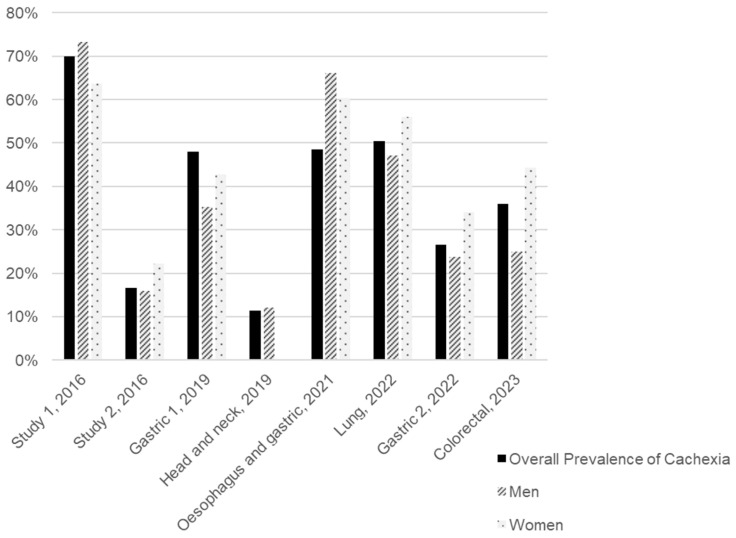
Overall and sex-specific prevalence of cancer-associated cachexia (CAC) in eight studies arranged in chronological order of publication. The tumour type assessed in each study is presented under each group of bars. Two studies included patients with different tumour types. Study 1 included patients with breast, GI tract, lung, and head and neck cancer, while study 2 included patients with colorectal, hepato-pancreato-biliary, skin/soft tissue, breast, and other cancers [20,21,22,23,24,25,26,27].

We conducted a review of the literature published over the last decade to estimate the distribution of CAC prevalence by sex and tumour type, identifying eight studies that presented these data, as illustrated in Figure 2 and Table S1. All the studies included were clinical trials involving cancer patients undergoing various types of treatment and of different ages (Table S1). Considering the subjects from all the studies included, the average prevalence of CAC is 31.5% for men and 42.9% for women. However, we must consider the disparity between studies, particularly in terms of sample sizes and the types of tumours included. Although the patient samples of these studies were predominantly composed of men (75.1%), and the types of tumours included (stomach, head and neck, oesophageal, lung, and colorectal) are more prevalent in men according to the Global Cancer Observatory (GLOBOCAN), as observed in Table 1, the prevalence of CAC was higher among women [19,20,21,22,23,24,25,26,27]. Figure 2 highlights a gender disparity in the sampled population, complicating the interpretation of CAC prevalence by sex.

Therefore, our review focuses on compiling information on the sexual dimorphism observed in pathways implicated in the development and progression of cachexia. Given the limited data on dimorphism specifically related to cachexia, we focused on pathways that are potentially sexually dimorphic, either under physiological conditions or in other well-characterised diseases. This highlights the imperative need for clinical studies to develop sex-specific treatments, where feasible, to enhance the effectiveness of interventions for the prevention or management of this debilitating condition.

## 2. Inflammation

CAC involves systemic inflammation mediated by molecules derived from tumours and host cells, particularly immune cells. These inflammatory mediators exacerbate catabolic and anorexigenic pathways, reducing food intake, causing negative nitrogen balance, and causing tissue disruption. Pro-cachectic inflammatory molecules include tumour necrosis factor alpha (TNFα), interferon gamma (IFNγ), and interleukins-1β, -6, and -8 (IL-1β, IL-6, and IL-8) [4,11,28]. A higher neutrophil-to-lymphocyte ratio (NLR) is linked to cancer progression and decreased survival [29,30]. Specifically, an inverse relationship between body weight and NLR was observed in a cohort of both male and female non-small cell lung cancer patients, indicating that NLR may be implicated in CAC development [31].

Particularly, TNFα and IL-1 are linked to skeletal muscle breakdown by activating nuclear factor-kappa B (NF-κB), and various pro-inflammatory cytokines downregulate key muscle proteins. A study in murine C-26 adenocarcinoma cells showed that TNFα and IFNγ together downregulate myofibrillar protein myosin heavy chain (MyHC) at mRNA and protein levels. TNFα also recruits neutrophils and macrophages to skeletal muscle and stimulates lipolysis in adipose tissue [4,32].

Furthermore, inflammation can activate the acute phase response, a complex process triggered by homeostatic disturbances, which can be harmful if excessively activated. In this process, the liver enhances the production of positive acute phase proteins, increasing the demand for amino acids. Additionally, there is a decrease in the circulating levels of negative acute phase proteins, such as albumin, not due to reduced synthesis, but rather due to increased microvascular permeability. Cytokines like TNFα, IFNγ, IL-1, and IL-6 are major promoters of the acute phase response, which increases muscle catabolism and resting energy expenditure, fostering cachexia. The quantification of C-reactive protein (CRP), a positive acute phase protein, is widely used to assess systemic inflammation and to predict survival and CAC progression [33,34,35].

**Figure 3:**
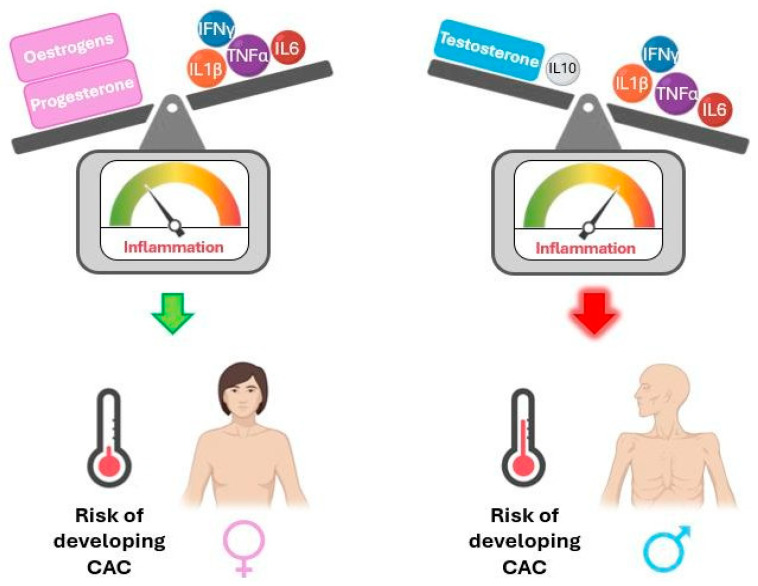
Interaction of sex hormones with cytokines influences the risk of developing cancer-associated cachexia (CAC) due to systemic inflammation. IFNγ: interferon gamma; IL1β: interleukin-1 beta; IL6: interleukin-6; IL10: interleukin-10; TNFα: tumour necrosis factor alpha. Interaction of sex hormones with cytokines influences the risk of developing cancer-associated cachexia (CAC) due to systemic inflammation. IFNγ: interferon gamma; IL1β: interleukin-1 beta; IL6: interleukin-6; IL10: interleukin-10; TNFα: tumour necrosis factor alpha. The balance of inflammation control is tilted towards the stronger part.

The literature indicates sexual dimorphism in inflammatory mediators across various physiological contexts. The following paragraphs focus on this evidence, which is summarised in Figure 3, and may help shed light on sex differences in cachexia, which is currently poorly understood.

TNFα, IL-1β, and IL-6 suppress testosterone secretion by acting on the hypothalamus and testicles, while testosterone supplementation in hypogonadal men reduces pro-inflammatory markers. Accordingly, older men show increased pro-inflammatory cytokines and decreased testosterone levels compared to younger men. Additionally, testosterone stimulates anti-inflammatory IL-10. Male newborns were observed to have reduced TNFα and higher IL-10 serum levels than female newborns, with higher testosterone but no significant difference in oestradiol levels in umbilical cord serum. These data support a relationship between androgens and inflammation, suggesting that testosterone may have immunosuppressive properties. On the other hand, women’s innate and adaptive immune responses are reportedly more robust than men’s [36,37,38,39,40,41].

However, the literature on sex differences regarding immunity is contradictory. Some studies observed that men display higher levels of pro-inflammatory cytokines and lower levels of anti-inflammatory cytokines (namely, IL-10) than women [42,43]. Particularly, men produce more monocyte-derived cytokines (IL-1β, IL-6, and TNFα), while women have more robust innate and adaptive immune responses and higher levels of lymphocyte-derived cytokines (such as IL-22). This may partly explain why men are more vulnerable to infection-related and cardiovascular diseases, whereas women are more affected by autoimmune diseases [40].

Conversely, oestrogen suppresses pro-inflammatory pathways, targeting inflammatory mechanisms associated with CAC, such as IL-6 transcription, IL-6-dependent CRP production, and TNFα and NF-κB signalling [44,45]. A study employing the *Apc*^Min/+^ mouse model of CAC concluded that males presented a higher degree of body weight loss, had higher levels of circulating IL-6, and had larger spleens compared to their female counterparts. These data indicate a higher level of inflammation in males, although there were no sex differences regarding tumour burden. Additionally, circulating IL-6 levels were not associated with cachexia onset and progression in females but were in males [46]. Another study from the same group revealed that cachexia and spleen weight in *Apc*^Min/+^ female mice were positively associated with the loss of oestrous cycling. Surprisingly, ovariectomy positively impacted multiple aspects of cachexia. However, upon IL-6 overexpression, ovariectomised mice experienced increased weight loss, thus suggesting that the loss of oestrogen production may increase females’ susceptibility to the effects of IL-6 [47]. Additionally, a study performed in the colon-26 adenocarcinoma mouse model observed that cachectic males displayed a stronger pro-inflammatory response (although not significant), accompanied by significantly enhanced loss of muscle and body weight, compared to their female counterparts [48].

Progesterone, the second major female hormone, is linked to anti-inflammatory outcomes. It reduces the activation of dendritic cells, diminishes their production of TNFα and IL-1β, downregulates NF-κB and toll-like receptor signalling, and reduces IFNγ production by natural killer cells. Progesterone also induces a shift from a pro-inflammatory helper T cell (Th1) to an anti-inflammatory Th2 phenotype [41].

Overall, it is likely that the more pro-inflammatory cytokine profile observed in men compared to women may contribute to the higher prevalence of severe muscle wasting and CAC in males compared to females [11,48]. However, it must be considered that oestrogen activity can either suppress or promote inflammation depending on the tissue and physiological context [49].

## 3. Neuroinflammation

Cancer patients often undergo several neurological alterations leading to anorexia, increased catabolic pathways, and, ultimately, the development of CAC [50]. Neuroinflammation frequently accompanies tumour growth and fuels cachexia, as hypothalamic inflammation magnifies systemic inflammation [4].

It has long been documented that IL-1 action on the hypothalamus increases the secretion of corticotropin-releasing hormone, a food intake inhibitor, and downregulates neuropeptide Y (NPY) expression, an appetite stimulant, promoting reduced ingestion [51,52]. IL-1β action on the hypothalamus also stimulates the hypothalamic-pituitary-adrenal axis, leading to increased release of glucocorticoids, which promote muscle proteolysis and adipose tissue breakdown. Furthermore, neuroinflammation impacts the central melanocortin system, which regulates the anorectic effects of serotonin. IL-1 and TNFα act at the hypothalamic level, promoting serotonin signalling and consequent NPY inhibition and muscle catabolism [4,53].

Myeloid cell migration to the central nervous system also plays a part in CAC development. A study conducted in a mouse model of pancreatic ductal adenocarcinoma found particularly high levels of neutrophils expressing C-C chemokine receptor type 2 (CCR2) in the brain of mice early in the disease development. Suppression of CCR2 reduced brain-infiltrating neutrophils and alleviated CAC by reducing anorexia and muscle catabolism. Additionally, blocking the purinergic receptor P2RX7 signalling on brain macrophages, implicated in neutrophil recruitment to the brain during neuroinflammation, attenuated immune cell infiltration into the brain and alleviated cachexia [54].

Growth and differentiation factor 15 (GDF15), lipocalin-2 (LCN2), and insulin-like 3 have been recently identified as factors that activate neuro-cachexia symptoms. LCN2 was found to be involved in appetite suppression during pancreatic CAC. LCN2 modulates gene expression in both appetite-suppressing pro-opiomelanocortin (POMC) neurons and appetite-stimulating NPY/AgRP neurons, affects immune cells, and upregulates endothelial inflammation genes and cachexia-specific oligodendrocyte genes in the hypothalamus [55,56]. Furthermore, several molecular signatures observed in the medial basal hypothalamus during cachexia are also found in the peripheral tumour microenvironment, such as CCR1+ macrophages expressing TNF, IL-1β, and IL-6 [55].

Macrophage inhibitory cytokine-1 (MIC-1), also known as GDF15, promotes tumourigenesis by stimulating metastasis, angiogenesis, inflammation, and tumour immune escape properties, among other aspects. Recently, glial cell-derived neurotrophic factor family receptor alpha-like (GFRAL) was identified as the MIC-1 receptor, and its expression has only been observed in the brain. MIC-1 has been linked to cancer anorexia by acting in the area postrema of the brain, and it has been suggested that MIC-1/GFRAL/ret proto-oncogene signalling contributes to CAC [50,57]. Serum MIC-1 concentrations were found to be four times higher in prostate cancer patients with cachexia compared to those without cachexia [58], and its expression was positively associated with the expression of the pro-tumorigenic M2 macrophages in prostate tumours [59]. Interestingly, androgens stimulate MIC-1 expression in the prostate, and MIC-1 overexpression is linked to the progression from androgen-sensitive to androgen-independent and metastatic prostate cancer [57,60,61]. It should be noted that cachexia is particularly prevalent among prostate cancer patients [11].

The gut–brain axis is also an important aspect of cachexia. Inflammatory mediators potentiate intestinal permeability, increasing the amount of circulating pathogen-associated molecular patterns (PAMPs), such as bacteria-derived lipopolysaccharide (LPS). PAMPs are then recognised by the hypothalamus, leading to the exacerbation of hypothalamic inflammation. Using two mouse models of pancreatic CAC, a study found that cachexia-inducing tumour secretomes, particularly prostaglandin E2, enhanced LPS-mediated hypothalamic inflammatory response. Moreover, the level of cachexia displayed by different mouse models correlated with the intestinal barrier dysfunction, highlighting the importance of the tumour–gut–brain signalling in CAC [62]. In another study, administering LPS in animal models of infection-associated anorexia increased NF-κB activity in the hypothalamus, a known CAC mediator. This upregulates IL-1β, IL-6, and TNFα in the medio-basal hypothalamus. Additionally, intraperitoneal injections of leptin and LPS led to an NF-κB-induced increase in pro-opiomelanocortin expression, a melanocortin precursor, in the hypothalamus [63].

To our knowledge, no studies have explored sexual dimorphism in neuroinflammation during CAC, but it has been examined in other conditions. A study found that LPS-induced inflammation in primary mouse astrocytes differs by sex, likely due to perinatal testosterone exposure. Male and androgenised female astrocytes had higher IL-6, TNFα, and IL-1β mRNA levels than females (*p* < 0.001), while IP10 levels were higher in LPS-treated females (*p* < 0.01) [64]. Another study observed that LPS injection triggered a greater inflammatory response in male and ovariectomised female rats compared to sham-operated females. Additionally, both ovariectomised and sham-operated females receiving LPS exhibited increased testosterone levels, with no significant differences in oestradiol, and oestradiol replacement decreased LPS-induced fever in ovariectomised females [65].

Velez et al. conducted an in-silico survey of human genetic correlations, predicting that hypothalamic inflammatory pathways induced by muscle-derived TNFα are stronger in males than in females [66]. A review reported that the exposure to unfavourable environmental conditions in utero, such as maternal stress and air pollution, resulted in increased expression of pro-inflammatory cytokines, such as IL-1β and TNFα, and decreased expression of IL-10 in the brain of male adults, which was not observed in females [67].

## 4. Adipose Tissue

Adipose tissue (AT) is crucial in CAC, impacted by lipolysis, suppressed adipogenesis, and in WAT browning. Beyond storing excess lipids, it plays an endocrine role by secreting hormones and adipokines that regulate appetite and nutrient metabolism. Changes in AT mass significantly impact overall energy balance. In CAC, inflammatory signals from tumours disrupt normal communication between AT and other organs, leading to energy imbalance and lipolysis [68,69]. In CAC, fat is lost more rapidly than lean tissue. This is partly due to reduced food intake and the effects of tumour factors and inflammatory cytokines like TNFα that inhibit lipogenesis or promote lipolysis [70,71,72,73,74,75]. AT is located in visceral white adipose tissue (vWAT) and subcutaneous white adipose tissue (sWAT) depots, each with distinct characteristics. The location of fat impacts metabolic processes, including hormone production, insulin sensitivity, inflammatory responses, mitochondrial function, varying gene and protein expression, and free fatty acid release.

vWAT contains large unilocular adipocytes, responds more quickly to nervous system-induced lipolysis, is linked to metabolic disorders, and is more abundant in men. In contrast, women store more sWAT, particularly post-puberty, which is associated with better insulin sensitivity and long-term lipid storage. Ageing alters the vWAT-to-sWAT ratio by increasing vWAT due to changes in cellular functions, including adipocyte generation, hormone release, and lipid handling. Women have more sWAT in the abdomen and gluteofemoral area, particularly in the superficial compartment. Women also typically have lower intra-abdominal/visceral fat, although this distinction diminishes with age [3,4,5,76,77,78,79,80,81,82]. The effects of testosterone on fat distribution appear to be the opposite of oestrogen. While oestrogen promotes fat storage in the lower body (femoral/gluteal regions), and its loss leads to increased visceral fat (as seen in postmenopausal women), testosterone seems to prevent lower-body fat accumulation. When testosterone levels are suppressed, men store more fat in the thighs instead of the usual abdominal distribution. This suggests that testosterone acts as a suppressor of lower-body fat storage, whereas oestrogen encourages it [83,84]. In obesity, male adipose tissue accumulates proinflammatory macrophages, contributing to insulin resistance, while females are generally protected. Obese women show a milder inflammatory response, which can intensify with lipolysis. Androgen receptors in adipose tissue macrophages may influence metabolic effects in men. However, interactions between sex hormones, immune cells, and adipose function are complex and context-dependent; hormones, immune cells like macrophages, and adipose tissue function are intricate and can vary based on the specific context, including genetic factors, hormonal levels, and overall health status [85,86,87].

The following paragraphs explore sexually dimorphic adipose tissue alterations in non-cachectic contexts, highlighting their dimorphic relevance in understanding cachexia.

### 4.1. Lipolysis

Research indicates that insulin plays a role in influencing lipolysis. It has been proposed that insulin, along with β-adrenergic signalling, exerts anti-lipolytic effects by activating PDE3B through PKB. Furthermore, at elevated concentrations, insulin’s suppressive impact on lipolysis is reversed, leading to a restoration of lipolytic activity [88]. While insulin typically exerts anti-lipolytic effects, at elevated levels, it can restore lipolytic activity by inhibiting hormone-sensitive lipase and facilitating triglyceride accumulation in adipocytes [88,89,90,91]. Furthermore, studies show that men have higher levels of lipoprotein lipase (LPL) activity and larger fat cells, which leads to higher basal lipolysis in men [90]. Additionally, cachexia is often associated with insulin resistance, removing its antilipolytic effect, which further promotes lipolysis [70,71,72,73,74,75].

Lipolysis also varies by fat storage site, adrenergic receptor types, and their abundance within each site [92]. Adrenergic beta-3 receptor stimulation triggers lipolysis in both sexes, but obese females show a more pronounced response. Testosterone boosts lipolysis in male adipose cells by increasing β-adrenergic receptors and adenylate cyclase activity, influencing fatty acid turnover and LPL suppression [85,93,94,95,96]. Men show lower lipolysis stimulation by noradrenaline in vWAT due to catecholamine resistance. Testosterone therapy in men with hypogonadism increases FA turnover and suppresses LPL, therefore increasing lean body mass, reducing fat, and improving metabolic parameters like glucose-insulin homeostasis [97,98,99,100]. Testosterone’s impact on lipolysis varies among studies, but some show it boosts the lipolytic ability of adipose precursor cells in male rats by increasing β-adrenergic receptor numbers and adenylate cyclase activity [93,94,96].

Oestrogen in females promotes preadipocyte proliferation, has anti-lipolytic effects, and upregulates adipose-browning genes in SAT while enhancing lipolysis in VAT. In males, oestrogen decreases energy production gene expression but reduces macrophage infiltration and inflammation in VAT. It also influences WAT gene expression, enhancing lipolysis and reducing lipogenesis, with local oestrogen production affecting metabolism [85,93,94,95,96]. The overall effect of oestradiol on adipose tissue is multifaceted. The increase in adiposity in post-menopausal women is an example that despite lacking this inhibitory effect of oestradiol on lipolysis, the adiposity is augmented. This process can have multiple explanations, such as a shift in fat storage distribution (sWAT to vWAT), a change in appetite, and compensatory mechanisms [97].

### 4.2. Lipogenesis

A key factor in the reduction of AT in cachectic patients is a decrease in lipogenesis, in which fatty acids are synthesised from glucose. This process begins with elevated blood glucose levels, triggering insulin release from the pancreas, facilitating glucose uptake by adipocytes, activating enzymes involved in glycolysis and lipogenesis, and enhancing gene expression necessary for lipogenesis. Glucose metabolism generates Acetyl-CoA, the building block for FA synthesis. FAs combine with glycerol to form triglycerides stored as an energy reserve in WAT [3,101]. Obese female mice have higher glucose tolerance, more adiponectin, and reduced immune cell infiltration and oxidative stress in adipose tissue compared to males, suggesting oestrogen’s protective effects. Oestrogen’s antioxidant properties and ability to induce longevity genes contribute to stress resistance and longer life expectancy in females, which may, in part, be attributed to metabolic variations, specifically molecular and functional differences in adipose tissue depots between sexes [102,103,104]. Elevated local oestrogens influence adipocyte differentiation, lipogenesis, and metabolic signalling. Hetemäki et al., in their research, concluded that visceral fat of hormone therapy users shows lower testosterone levels and a stronger response to oestrogen therapy compared to the subcutaneous one [82].

### 4.3. WAT Browning

Brown adipose tissue (BAT) significantly impacts systemic metabolism beyond energy expenditure and body fat regulation. In the context of CAC progression, the browning process of adipose tissue occurs in early stages. Although WAT mainly serves as an energy reservoir, brown adipocytes also store TAG but accelerate lipid breakdown by upregulating UCP-1. This process boosts the release of inflammatory peptides, drawing in stromal cells, macrophages, and lymphocytes, which significantly reshape the adipose tissue environment. Several factors contribute to the browning process. These include central nervous system control, transcription factors (PPARγ, PGC-1a, PDRM16, C/EBP-b), and proinflammatory cytokines (IL-6, TNFα) [105,106,107].

Studies show BAT transplantation improves glucose tolerance and insulin sensitivity in mice. In humans, higher BAT activity correlates with lower blood glucose and HbA1c levels [108]. In brown adipocytes, ERα is the main receptor, while ERβ supports browning and has beneficial effects against obesity, diabetes, and lipid accumulation [109,110,111]. Women have higher BAT activity than men. Oestrogens enhance BAT activity via suppressing alpha 2 adrenergic receptor transcription and increasing the sensitivity to sympathetic signalling, while androgens inhibit it. Ovariectomy decreases BAT thermogenic activity and uncoupling protein 1 (Ucp1) mRNA expression, but E2 administration reinstates these levels [109,111].

Progesterone’s effects on BAT are unclear. In vitro studies show that progesterone either inhibits or stimulates Ucp1 mRNA expression and lipolysis, depending on varying concentrations. Androgen levels affect BAT differently in men and women. Hyperandrogenism in women, like in polycystic ovarian syndrome, reduces BAT activity and increases abdominal obesity, while hypogonadism in men leads to obesity. In vitro studies show that androgens inhibit mitochondrial biogenesis, brown adipocyte differentiation, and norepinephrine-induced lipolysis. Testosterone and dihydrotestosterone inhibit Ucp1 mRNA expression and mitochondrial respiration [109,111]. Testis removal in animals increases UCP1 expression, aligning with findings in women with hyperandrogenism. Both sexes experience a decline in BAT activity with age, but women preserve higher activity [95,108,112,113,114].

**Figure 4:**
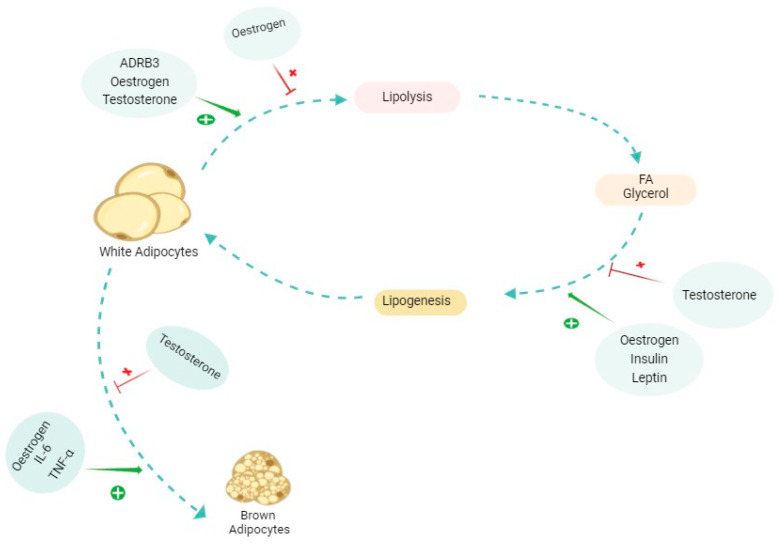
The effects of different molecules and hormones on the alterations of adipose tissue (AT). The figure shows a simplified promoting (shown in green plus) and inhibiting (shown in red cross) effect on each of the pathways involved in adipose tissue alterations, whether in cachexia or physiological conditions. The effects of each of these molecules are complex and multifaceted and differ in various contexts. Oestrogen specifically has dual effects on lipolysis depending on the type of AT and in each sex. ADRB3: beta 3 adrenergic receptor; FA: fatty acid; IL-6: interleukin-6; TNFα: tumour necrosis factor alpha.

Figure 4 shows a summary of some major elements involved in the AT alterations, and Table 2 shows a summary of sexually dependent hormones affecting AT.

## 5. Muscle

During CAC, muscle tissue becomes a source of energy via catabolic processes that release amino acids. Furthermore, the inhibition of anabolic pathways leads to muscle loss [4,122]. One of the primary catabolic pathways upregulated in CAC is the ubiquitin-proteasome system (UPS), which plays a crucial role in promoting protein degradation [122,123,124,125]. Increased autophagy also correlates with muscle atrophy and energy wastage during CAC [3,126,127].

The involvement of the calcium-activated calpain protease pathways adds another layer of complexity to CAC. This pathway involves the cleavage of myofibrillar proteins from the sarcomere for subsequent proteolysis by other systems, mainly the UPS, which is overexpressed during CAC [3,122,128,129]. Pathways involved in protein catabolism during CAC, such as the UPS and autophagy, appear to be influenced by sex. Women generally have lower basal UPS activity, partly due to oestrogen signalling, while men exhibit less protein degradation via autophagy [130,131,132]. In CAC, resistance to anabolic pathways is also experienced. Insulin-like growth factor 1 (IGF-1), which promotes protein synthesis and inhibits muscle degradation under homeostatic conditions, is downregulated during CAC [4,122,133]. Additionally, insulin, an anabolic hormone that stimulates glucose uptake by muscle and adipose tissue, is affected, leading to increased protein degradation and subsequent muscle loss. This may be associated with the chronic inflammatory state of patients, resulting in metabolic imbalances, including pancreatic β-cell dysfunction and impaired insulin secretion. Cachectic patients exhibit reduced circulating levels of IGF-1 and peripheral resistance to GH and insulin, impairing the amino acid balance in skeletal muscle [70,122,134,135,136]. Sexual dimorphism related to IGF-1 and insulin resistance in CAC is still poorly understood.

Secreted by muscle cells, myostatin increases protein degradation, decreases protein synthesis, and impairs the activation of satellite cells as well as the proliferation and differentiation of myoblasts. Myostatin activates NF-κB signalling and stimulates the UPS [9,122,137,138]. Satellite cells, which are myogenic stem cells, become activated and proliferate to form new myonuclei, thereby facilitating muscle hypertrophy or regeneration following acute or chronic injuries [130,139,140]. There are differences between male and female satellite cells. Men appear to have higher satellite cell content, more differentiation- and hypertrophy-related mRNAs, such as myogenin and MyoD, and a greater proliferative capacity than women [130,139,141]. In skeletal muscle, the androgen receptor is expressed by satellite cells, and testosterone mediates more excellent satellite cell content in men, possibly allowing for greater muscle size [130,141]. Women tend to have a smaller number of satellite cells due to their activation by testosterone through androgen receptors. However, oestrogen appears to have a protective effect on satellite cells during CAC, acting on ERα and β receptors, which are also present in these cells [136,142,143].

### 5.1. Mitochondrial Metabolism

Mitochondrial dysfunction in CAC encompasses reduced ATP synthesis, uncoupling of oxidative phosphorylation, decreased oxidative capacity, disrupted protein synthesis, altered membrane fluidity, and oxidative modification of mitochondrial proteins [9,50]. Mitochondria can respond to muscle signals by remodelling their morphology through mitochondrial fusion and fission. The expression of the fusion proteins MFN1 and MFN2 decreases during CAC, while the expression of the fission protein FIS1 increases. Mitophagy also appears to rise in CAC [122,144,145,146].

Human muscles are composed of three types of fibres: type 1 fibres (slow or oxidative), type 2A fibres (intermediate or oxidative-glycolytic), and type 2X fibres (fast or glycolytic) [142]. Men’s muscles generally have a greater abundance of type 2X fibres, which directly affects glucose metabolism and respiratory capacity, and these fibres are less resistant to fatigue than those of women. In contrast, women have a higher proportion of type 1 muscle fibres and rely more on oxidative metabolism [130,139,142]. CAC affects glycolytic fibres more strongly than oxidative fibres, which could partially explain why CAC affects men more than women [130,139]. Women have greater intrinsic respiratory capacity and mitochondrial content in their mitochondria, suggesting that women have greater mitochondrial quality than men, favouring a more oxidative profile. Additionally, women have more type 1 muscle fibres, which contain more mitochondria [112,139,142]. An RNA sequencing study of skeletal muscle in men and women showed a significant transcriptomic difference between the sexes. Genes related to mitochondrial function were more expressed in women, while genes related to protein catabolism were highly expressed in men [147]. Another study performed on rat gastrocnemius observed that female rats had significantly more mitochondrial and protein content, as well as more mitochondrial DNA per gram of tissue, more TFAM protein, and more mitochondrial complexes than males [148].

Sex hormones play a crucial role in mitochondrial function. Through its receptor, oestrogen can directly modulate the expression of critical genes for mitochondrial function. Furthermore, in association with its receptor, oestrogen triggers a phosphorylation cascade and limits mitochondrial oxidative damage [112,130,149]. In ovariectomised rats, oestrogen deficiency affected mitochondrial health, leading to a significant increase in peroxide production. However, when these rats were given oestrogen replacement shortly after ovariectomy, mitochondrial efficiency was significantly preserved, but when oestrogen replacement occurred a few weeks later, its beneficial effects disappeared [150]. Progesterone appears to have receptors on mitochondria that can directly increase mitochondrial respiration and muscle efficiency [130,151]. Although little is known regarding testosterone, low testosterone levels in men appear to be associated with reduced mitochondrial gene expression and respiratory activity [146].

Oxidative phosphorylation is also affected by mitochondrial dysfunction in CAC, leading to muscle loss. There is a decrease in the expression levels of OXPHOS subunit proteins, impairing mitochondrial activity, and an upregulation of uncoupling protein 2 and 3 genes, promoting inefficient ATP synthesis. The activity of SERCA (Sarcoplasmic Reticulum/Endoplasmic Ca^2+^-ATPase), an enzymatic complex essential to muscle function, is increased in CAC, promoting energy inefficiency. Peroxisome proliferator-activated receptor gamma coactivator 1-alpha (PGC-1), a protein that regulates energy metabolism and mitochondrial biogenesis, is reduced during CAC, resulting in the loss of mitochondrial content and ATP production in muscle [3,9,122,145]. A study demonstrated that functional mitochondria degeneration precedes muscle loss in CAC progression [152].

### 5.2. Muscle Structure and Mass

Muscle mass is influenced by sex in CAC. Although men physiologically have more muscle mass than women, in CAC, men typically experience greater muscle loss and a higher incidence of sarcopenia compared to women [139,141,142]. In a study involving patients with locally advanced or recurrent non-small cell lung cancer, men showed a proportionally greater reduction in muscle mass and were classified as sarcopenic, whereas women were not [153]. Similarly, in another study where cancer patients were followed during the last 2 years of life, the prevalence of muscle wasting was significantly higher in men than in women [154]. A study of Lewis lung carcinoma using a mouse model showed sex-specific gene expression patterns. Females showed downregulation of interferon-associated genes, supporting the preservation of muscle mass. In contrast, males showed elevated interferon signalling, significant deficiencies in energy metabolism pathways, and loss of muscle mass [155,156]. Furthermore, the more pronounced muscle loss in males may be related to hypogonadism and the higher prevalence of lung and gastrointestinal cancers that most commonly result in CAC [139,141,142,157,158].

Fatigue and quality of life in CAC differ by sex. Cachectic men have significantly less strength, power, muscle mass, and muscle quality than healthy men, with a lower quality of life and greater fatigue. Cachectic women also exhibit significantly reduced strength and muscle quality compared to healthy women; however, their quality of life and fatigue scores remain similar [159]. In a study of patients with aggressive B-cell lymphoma, men with sarcopenia had significantly reduced progression-free survival and overall survival compared to non-sarcopenic men. In contrast, sarcopenic women showed no difference in progression-free survival but exhibited a trend toward increased overall survival compared to non-sarcopenic women [160]. The available evidence for understanding sexual dimorphism in muscle during CAC is limited, as most studies are conducted in men or do not consider this aspect [130,158].

**Figure 5:**
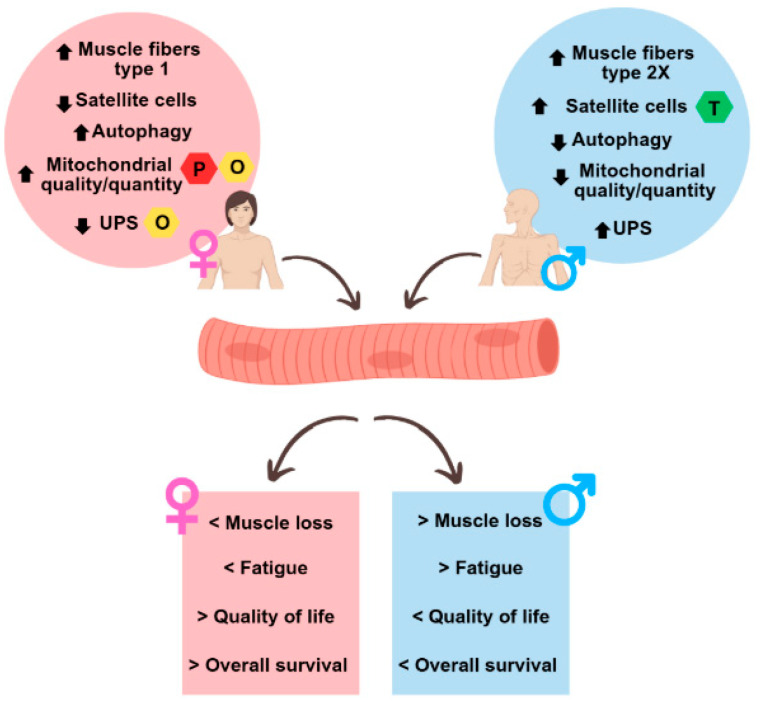
Summary of sex differences observed in the muscles of cachectic cancer patients. The upper circles represent sex differences in muscle at the molecular level under normal physiological conditions and in cancer cachexia, while the lower squares represent the different outcomes between men and women in cancer cachexia. UPS: ubiquitin-proteasome system; O: oestrogen; P: progesterone; T: testosterone.

Figure 5 highlights the major sex differences in muscle physiology in healthy conditions and during CAC.

## 6. Gastrointestinal Tract

Another key feature of CAC is gastrointestinal tract malfunction, resulting in gastrointestinal barrier dysfunction. The main reason behind the elevated gastrointestinal barrier dysfunction during CAC remains unidentified but could involve factors such as tumour expansion, inflammation, and mucosal harm. Elevated levels of IL-6 are linked with both gastrointestinal barrier dysfunction and cachexia, indicating that impaired barrier function might also affect other forms of cancer. Moreover, alterations due to cytotoxic agents or radiotherapy are associated with gastrointestinal issues such as diarrhoea, mucositis, and increased susceptibility to infections [161,162]. Another notable element, especially prevalent among those with GI cancer, is malnutrition. Chemotherapy-related symptoms like decreased appetite, nausea, vomiting, and changes in the microbiome that serve as both an example and a contributing factor to intensifying the malnutrition in CAC [163].

### 6.1. Ghrelin

Ghrelin, predominantly produced in the stomach, is pivotal in appetite and energy control, signalling hunger to the brain. The secretion of ghrelin follows a pulsatile pattern and is affected by various factors such as insulin, leptin, and noradrenaline. It modulates food consumption, energy use, fat storage, and muscle loss by stimulating GH secretion and through other pathways. Increased ghrelin levels in CAC patients might serve as a compensatory reaction to tumour-driven appetite loss [164,165]. Research shows that female rats demonstrate notably elevated levels of acylated ghrelin in their bloodstream and reduced hepatic expression of the ghrelin receptor antagonist LEAP-2 compared to male rats. Additionally, females exhibit greater expression of the ghrelin receptor (GHSR1A) in brain regions associated with the regulation of anxiety and feeding behaviour [166]. Ghrelin has notable anti-inflammatory effects, suppressing pro-inflammatory cytokines such as IL-1β, IL-6, and TNFα while inhibiting NF-κB signalling to reduce inflammation. Additionally, it plays a role in modulating hypothalamic feeding circuits during stress, particularly in females. Anamorelin, a ghrelin receptor agonist, has a longer half-life than ghrelin and is effective in mitigating muscle and fat loss in cancer patients. Its therapeutic benefits extend to improving appetite and lean body mass, making it a valuable treatment for conditions like cancer cachexia [167,168,169]. Kan et al. reported a case of pancreatic cancer (male patient) in which the patient exhibited a remarkable response to anamorelin, an orally active ghrelin receptor agonist, leading to significant weight gain [170].

### 6.2. Gut Microbiota

The collection of bacteria, archaea and *eukarya* living in the gut is referred to as the ‘gut microbiota’. Throughout years it has evolved alongside the host, forming a symbiotic connection [171,172]. The strong connection between the gut microbiota and the host often leads to changes in the composition of gut bacteria, known as dysbiosis. This shift is commonly seen in health conditions, including gastrointestinal diseases and neurodevelopmental disorders. These variations could also contribute to CAC, as diverse bacterial species possess varying capabilities to generate nutrients and inflammatory substances, potentially leading to the deterioration of muscle and fat tissues [171,173,174,175]. A key role of the gut microbiome is to coordinate the two-way communication between the gut and the brain, enabling the integration of immune, metabolic, and endocrine signals from both peripheral and central sources. There are significant sex differences in the downstream effects of this axis, particularly within the neuroendocrine and neuroimmune systems [176]. Studies show alterations in gut microbiota can both directly and indirectly affect the gonadal activities. This can occur either via metabolic signals that initiate puberty, leading to sex-specific developmental processes, or through alterations in the gut microbiota that affect gonadal hormones and neurotransmitters, influencing brain development [176,177]. The gut microbiota significantly impacts cancer-related cachexia by modulating bile acid metabolism, which influences critical processes like inflammation, metabolism, and muscle degradation. In cachectic mice, a decline in microbial 7α-dehydroxylation activity results in reduced levels of essential secondary bile acids, such as deoxycholic acid (DCA), known for their anti-inflammatory and metabolic regulatory roles. Furthermore, a decrease in gut-derived oxo-bile acids (7-oxoLCA, 12-oxoLCA) may contribute to weakened gut barrier integrity, a hallmark of cachexia [178].

In their study, Flores et al. observed that there exists a substantial and direct correlation between the diversity of species within the faecal microbiome (referring to the number of distinct species) and the systemic oestrogen levels [179]. Additionally, other studies indicate that female mice possess higher levels of beneficial bacteria like *Akkermansia*, promoting healthier lipid profiles and improved metabolic health. In contrast, male mice exhibit decreased gut microbiota diversity and different microbial responses to a high-fat diet, leading to dyslipidaemia and metabolic disorders [180,181]. Furthermore, both oestrogen and testosterone have different effects on immune cells in the gut. It is known that oestrogen, specifically β-oestradiol, transforms dendritic cells to produce IL-12 and IFN-γ, activating pro-inflammatory cytokine pathways. It also prolongs B cell survival and activates polyclonal B cells, creating a pro-inflammatory environment that increases gut permeability and promotes microbiota migration into the lamina propria, furthering inflammation. Conversely, testosterone inhibits T-cell proliferation without affecting the intestinal barrier. Blood testosterone levels correlate with gut microbiota, and castration in males reverses these sex differences, confirming androgen effects on the microbiome [182].

### 6.3. Absorption

Sexual dimorphism and the digestive system’s absorption processes are influenced by several factors. These include nuclear receptor-mediated signalling pathways throughout the digestive tract, sex-specific genes in the salivary glands, hormones that regulate appetite, and motility. Studies have shown that women have a sex-dependent protection against *H. pylori* infection and gastric lesions compared to men, a distinction not observed in children. Additionally, men show both a higher risk of developing gastric cancer and worse clinical outcomes once diagnosed [183,184,185]. Bile acid secretions are also reported to have higher concentrations of cholic acid in males and higher concentrations of chenodeoxycholic acid in females. Females generally take longer to digest food and have lower postprandial acid secretion compared to males. Gastroparesis is also four times more prevalent in females [186,187,188].

Furthermore, Soni et al. observed sexual dimorphism in GI mobility and permeability in malnourished mice. They concluded that female malnourished mice experienced delayed gastric emptying and no significant increase in gut barrier permeability, while male malnourished mice increased baseline contractile activity and heightened responses to cholinergic stimulation in duodenal segments and increased gut barrier permeability. These changes could be due to sex hormones, evolutionary adaptations, and differences in gut microbiota [189].

### 6.4. Liver

The liver regulates the metabolic rate by overseeing the transport, storage, and breakdown of glucose and lipid utilisation. Inflammation triggers it to produce acute phase proteins, causing muscle degradation into amino acids. In cachexia, heightened pro-inflammatory cytokines trigger lipolysis and muscle wasting and disrupt glucose metabolism [190,191,192]. 

The liver plays a substantial role in cancer-induced inflammation, with CRP acting as a vital prognostic indicator. Furthermore, impaired oxidative phosphorylation efficiency in the liver contributes to heightened metabolism during cancer [193]. Global gene expression analyses in mouse and rat livers have revealed over a thousand sex-specific transcripts. These transcripts significantly influence hepatic physiology, inflammatory responses, disease states, and the metabolism of steroids, lipids, drugs, and environmental chemicals. Sexual dimorphism is a crucial factor in liver metabolism, and its primary regulation involves the signalling of testosterone and oestradiol, as well as the sex-dependent expression of GH [194,195,196]. Sex-related differences in liver pathologies remain unclear. Some studies overlook sex as a factor, but evidence shows post-menopausal women may have a higher prevalence than men. Oestrogen influences lipid metabolism and adipose tissue regulation, with reduced signalling leading to liver fat accumulation. Oestrogen replacement therapy in postmenopausal women shows protective effects, involving ERα regulation and pathways like Small Heterodimer Partner and microRNA mir-125b [197,198,199]. The protective effect of oestrogen on the liver can also be seen in patients with pancreatic ductal carcinoma, where female patients are less likely to develop liver metastases; meanwhile, male patients exhibit higher expression of genes that promote hepatic metastasis compared to female patients [200].

Hepatocellular carcinoma (HCC) is more prevalent in males due to hormonal influences. Oestrogen and ERα provide protective effects, while androgens promote cancer progression. IL-6 plays a crucial role in sex differences in HCC development, with oestrogen inhibiting its production. FOXA1 and FOXA2 are essential for regulating these processes, highlighting the complex interplay between sex hormones and liver cancer [17,201]. Furthermore, administering oestrogens suppressed chemically induced hepatocarcinogenesis in males, and ovariectomy had a promoting effect on tumourigenesis. In the absence of ERα, females lost their protection against HCC; moreover, a combined oestrogen and progesterone regimen has been observed to reduce the risks of liver and thyroid cancers in post-menopausal women [115,202].

### 6.5. Drug Metabolism

Responses to different drugs can be sexually dimorphic, as they depend on sexually dimorphic parameters such as body weight, height, body surface area, fat mass, and plasma volume, which also depend on other factors such as genes, age, and hormones [116]. Diversity in enzymes responsible for metabolising the drugs is another factor that leads to this dimorphism between men and women. The difference in Cytochrome P450 (CP450) and its subfamilies plays an important role in this heterogeneity [117,118,119]. CP450, subfamily 2, polypeptide 11 (CYP2C11) and CYP2C13 exhibit higher constitutive expression levels in male rats compared to females, whereas CYP2C12 is notably more highly expressed in females. These enzymes play a part in protecting against drug toxicity by influencing drug metabolism pathways. These sex differences in gene expression translate into variations in drug metabolism rates for certain drugs [120,121]. Chemotherapeutic agents, metabolised in the liver, are affected by diversities in CP450 subfamilies. Paclitaxel, as a treatment for various cancers, is predominantly metabolised by liver enzymes, primarily through CYP2C8 and, to a lesser extent, CYP3A4. Studies have shown that male patients have a significantly better capacity to eliminate paclitaxel than female patients, with men demonstrating a 20% greater elimination volume [200,203]. Olaparib is a medication used to treat ovarian cancer, breast cancer, pancreatic cancer, and prostate cancer. It is mainly metabolised by CYP3A4/5 enzymes, which are expressed at higher levels in women compared to men [200,204].

### 6.6. Growth Hormone

Many sex-biased genes are regulated by GH, impacting lipid and drug metabolism pathways similarly in human and rodent liver [194]. The sexual dimorphism caused by differences in GH secretion is not solely due to the distinct patterns in males and females. In males, GH is secreted in pulsatile peaks every 3–4 h, whereas in females the peaks occur more frequently, approximately every 1.5 h, leading to a higher average GH concentration in females. GH binds to its receptor, activating the JAK2-STAT5b signalling pathway, which is essential for regulating sexually dimorphic gene expression. Additionally, this dimorphism is influenced by the differing responses of liver P450 expression to GH [117,205,206,207]. In their study examining the impact of GH signalling pathways on lifespan and cancer incidence using mouse models, Chhabra et al. found that wild-type female mice had a higher incidence of lymphoma than males. They also discovered that removing GH-STAT5 signalling decreases this incidence. Furthermore, the study indicates that GHR-knockout males have a median lifespan comparable to WT mice, while females demonstrate increases in both median and maximum lifespan [208].

Table 2 summarises some important factors for hepatic metabolism which have been linked to sexual dimorphism.

## 7. Menopause and Hormone Replacement Therapy (HRT)

Ageing and menopause are characterised by an abrupt loss of oestrogen production, leading to increased inflammation, muscle and bone loss, among various other molecular and physiological alterations [209,210]. Consistently, the risk of cancer increases with age, and, apparently, so does cachexia [211,212]. In mice, ageing was found to modulate CAC in a strain- and tumour-dependent manner (which may also occur in humans), and the reliability of inflammatory cytokines as cachexia biomarkers in patients varies with age [212]. Cyr, B. et al. reported that female mice exhibit age-induced inflammation at a higher level than males, probably due to the more pronounced decline in sex hormones experienced by females [213]. Moreover, menopause-related changes in gut microbiota (particularly reduced butyrate production) may negatively impact skeletal muscle mass in menopausal women [214].

In vitro E2 treatment reduced the expression of IL-1β, IL-6 and TNFα in different human cell types, including haematopoietic and immune cells. Additionally, HRT in postmenopausal has been shown to reduce circulating IL-6 levels [215]. Another study conducted on postmenopausal women with rheumatoid arthritis (a disease associated with cachexia) observed that upon treatment with E2 during 2 years, patients presented decreased soluble IL-6 receptors and increased IGF-1 [216].

In KPC mice, CAC developed earlier and more intensely in males compared to females in terms of muscle atrophy and fat mass wasting (this difference was neutralised in the later stages of the disease). In mouse myotubes treated with KPC-conditioned medium and oestradiol, the reduction in myotube diameter was mitigated, and gene expression of activin inhibitor Fstl1 was significantly enhanced, compared to KPC-conditioned myotubes. This indicates a putative protective role of oestradiol in pancreatic cancer cachexia. Muscle biopsies from women presented significantly higher expression of some activin inhibitors compared to men in patients with resectable pancreatic ductal adenocarcinoma. Moreover, male patients with advanced pancreatic ductal adenocarcinoma undergoing chemotherapy exhibited significant muscle wasting, whereas female patients did not [217]. Another study found that E2 administration in the cancer cachexia *Apc*^Min/+^ female mouse model reduced body weight loss and increased food intake and grip strength compared to both intact and ovariectomised tumour-bearing mice in a significant manner. Furthermore, mice under E2 treatment presented diminished phosphorylation of skeletal muscle AMPK (a protein highly associated with cachexia), increased expression of downstream targets of mTORC1 signalling (which is suppressed in cachexia), and improved cage activity and mitochondrial respiratory control ratio, compared to intact tumour-bearing mice [218]. However, it is important to note that ER levels and functionality decrease with age [219,220] and that oestrogens can promote the development of certain types of cancer, such as breast and gynaecologic cancers, in which HRT is not an option [49].

## 8. Therapeutic Options and Prognosis

### 8.1. Hormonal-Related Therapies

Progesterone analogues, such as megestrol acetate and medroxyprogesterone acetate, have been shown to moderately enhance appetite and increase body weight, primarily by promoting fat accumulation. Their effects are dose-dependent but temporary, as weight gain reverses once the medication is discontinued [221,222]. Furthermore, according to the latest rapid update of the ASCO guideline, in cases that patients cannot tolerate low-dose olanzapine, a short-term trial of a progesterone analogue or corticosteroid may be an alternative option for managing weight and appetite loss [223]. Among both male and female metastatic nasopharyngeal cancer patients with cachexia, administration of progesterone analogues significantly reduced circulating Epstein-Barr virus DNA, increased body weight, improved pain control, and improved overall quality of life [224].

Hypoandrogenism is also associated with cachexia symptoms in male patients with advanced cancer. Anabolic-androgenic steroids have demonstrated effectiveness in improving muscle mass and strength in individuals experiencing wasting due to acquired immune deficiency syndrome. A study administered testosterone replacement therapy (TRT) to male patients with hypogonadism and advanced, non-curative cancer. After 12 weeks of treatment, those patients presented lower serum TNFα (*p* < 0.005), higher IGF-1 (non-significant) and no changes in IL-6 compared to the group of patients not receiving testosterone [225,226]. As mentioned in the inflammation section, TRT produces anti-inflammatory effects. Specifically, testosterone decreases the production of pro-inflammatory cytokines (such as TNFα, IL-6, IFNγ, and IL-2) by inhibiting NF-κB signalling and group 2 innate lymphoid cells. Furthermore, testosterone fosters anti-inflammatory IL-10 production by activating the androgen receptor on CD^4+^ T lymphocytes [210]. The reduction of testosterone with age has detrimental effects on anabolic pathways in both men and women. A study observed that testosterone administration in healthy elderly men improved muscle wasting (*p* = 0.0001) while decreasing fat mass (*p* = 0.02) [211,227]. A recent review of the literature focused on the effects of TRT on cachexia. While some of the studies included reported no efficacy of TRT in ameliorating cachexia, others suggest potential benefits of this approach [228]. Adjunct TRT in male and female patients with advanced cervical or head and neck cancers resulted in an increased lean body mass (95% CI, −0.5–6.7%, *p* = 0.06) with no impact on survival. Sex differences in the outcomes were not evaluated [229]. Nevertheless, androgen replacement is not suitable for every type of cancer. Indeed, androgen deprivation therapy is frequently used to treat hormone-sensitive prostate cancer. This therapy has been associated with various adverse effects related to cachexia, such as sarcopenia, neuroinflammation, cognitive disorders and loss of energy [230,231]. Selective androgen receptor modulators, particularly enobosarm, offer a potential alternative to testosterone therapy for cancer cachexia and age-related sarcopenia due to their anabolic benefits with fewer side effects. The Prevention and Treatment of Muscle Wasting in Cancer Patients trials, clinical studies, showed that enobosarm improved muscle mass, stair climb ability, and quality of life in cachexic non-small-cell lung cancer patients. However, inconsistent functional improvements in later trials prevented further official approvals despite its positive effects on body composition [232,233].

### 8.2. Patient Care and Psychophysiology

Effectively managing cancer cachexia necessitates a holistic and individualised approach that targets both nutritional deficits and metabolic imbalances. The treatment strategy should emphasise improving food intake, minimising catabolic processes, and promoting anabolic pathways through a combination of dietary interventions, physical activity, pharmacological therapies, and psychological support. Additionally, ensuring symptom control and enhancing the patient’s overall quality of life remain fundamental aspects of care [226,234,235]. Individuals with CAC experience depression at nearly twice the rate of other cancer patients (30.2% versus 15.2%). Similarly, anxiety affects cachexia patients more frequently (18.6% compared to 11.1% in non-cachexia patients). Inflammatory processes may link these conditions. Specifically, elevated high-sensitivity C-reactive protein (hsCRP) correlates with both cachexia development and depression [236]. In the mouse model of male C57BL/6J mice that were injected with Lewis Lung Carcinoma cells, inflammatory molecules such as TNFα and IL-6 showed a crucial role in triggering various secondary symptoms that affected nutrition and mental wellbeing in cachexia. These include decreased appetite, discomfort, digestive issues, depression-like behaviour, and anxiety. Furthermore, hypothalamic-pituitary-adrenal axis, serotonin dysregulation, and vagal-afferent activation are among the alterations that trigger the secondary symptoms in CAC [237,238]. Evidence indicates that people with stronger cognitive abilities were more likely to recover from probable sarcopenia and return to a non-sarcopenic state. The progression of sarcopenia over time was more common in older men than in women [239]. Research on low back pain patients reveals interesting gender-based differences in pain-related beliefs and behaviour. Men, despite typically reporting less pain overall than women, tend to develop stronger avoidance behaviours specifically related to pain-inducing activities. This creates a notable contrast with general anxiety patterns, where women typically exhibit more anxious behaviours in medical contexts [240]. Lu et al. concluded that depression affects men and women differently at the molecular level, especially in the hypocretin system. For instance, they found that female depression patients had increased levels of hypocretin-1 in the hypothalamus, a brain area involved in emotion and arousal, but no such increase was seen in males [241]. Women experience higher rates of depression than men, with varying vulnerability levels. Oestrogen appears to play a central role in influencing both the development of major depressive disorder and the response to treatment. Fluctuating oestradiol levels have been linked to changes in fear-related behaviours, and clinical evidence suggests that premenopausal women respond more favourably to serotonergic antidepressants compared to men [242]. Response to psychiatric medications is also sexually dimorphic. As earlier mentioned, the sexual dimorphism in drug metabolism. For instance, depressed patients, male patients responded significantly better to imipramine than premenopausal females, but the females responded better to fluvoxamine than the males [243].

Individuals affected by CAC and their families should undergo regular assessments to ensure early detection of any psychosocial challenges or emotional distress [226]. Cognitive symptoms are also a concern in patients with CAC. For example, modafinil has been linked to improved memory and focus in breast cancer survivors. Additionally, donepezil has shown potential benefits in managing cancer-related fatigue and cognitive issues. Donepezil may help slow cognitive decline in individuals with brain metastases undergoing radiation therapy [244]. These therapeutic findings highlight the broader importance of addressing psychological and cognitive burdens in CAC, whether these challenges arise from the biological effects of the disease or the emotional strain of chronic illness. Moreover, the psychological impact of CAC may differ between sexes. Recognising these sex-specific responses allows for more personalised care. For instance, interventions targeting fear-avoidance behaviours may be particularly relevant for men, while women might benefit from approaches that emphasise different dimensions of symptom management, including pain and emotional coping strategies [240].

## 9. Conclusions and Future Perspectives

Cancer-associated cachexia has been increasingly recognised as a clinically significant syndrome and is a growing area of research. Several hallmarks of CAC have been established, involving multiple mechanisms across various organs. Despite both sexes experiencing functional and metabolic decrements due to cachexia, most studies on CAC in preclinical models and cancer patients have either not considered sex differences or have focused exclusively on males. The reluctance to study females is often attributed to concerns about the higher metabolic variability associated with the hormonal cycle. The sexually dimorphic nature of drug metabolism, gut microbiota, adipose tissue, and muscle, together with the protective effects of oestrogen against some diseases, highlights the importance of recognising and understanding sex differences in CAC. Men experience greater muscle mass loss and report worse quality of life and fatigue scores than women, which correlates with lower overall survival rates in cachectic men. Therefore, it is crucial to account for sexual dimorphism and sex hormones in all aspects of research. More studies involving females are needed to thoroughly understand hormonal complexities. Treatment guidelines for CAC and cancer should move towards precision medicine tailored to each patient to achieve better outcomes. Hormone profiling may be particularly beneficial for patient follow-up given their substantial influence.

## Figures and Tables

**Table 1:** Prevalence of cancer types stratified by sex, GLOBOCAN 2022 estimate [19].

Type of Cancer	Men (%)	Women (%)
Stomach	713,747 (65.5)	375,471 (34.5)
Head and neck	710,461 (75.0)	236,750 (25.0)
Oesophagus	531,019 (75.8)	169,226 (24.2)
Lung	1,572,045 (63.4)	908,630 (36.6)
Colorectal	2,641,491 (60.0)	1,765,609 (40.0)

**Table 2:** Some frequently discussed factors linked to sexual dimorphism responsible for hepatic alterations and metabolism. IL-22: interleukin-22.

Name	Role in Liver	Other Features
IL-22 [115,116,117,118,119]	Anti-inflammatory	Sex-dependent hepatoprotective
	Protective in liver injuries Promoting liver regeneration [115,116,117,118,119]	Higher levels in females than males [115,116,117,118,119]
Aquaporin 9 [120]	Responsible for anticancer drug uptake [120]	Sex-dependent expression [120]
BCRP (Breast Cancer Resistance Protein) [121]	Involved in biliary excretion, correlating with sex differences in pharmacokinetics of substrates like topotecan and doxorubicin [121]	Higher expression in male liver [121]

## Supplementary Materials

The following supporting information can be downloaded at: https://www.mdpi.com/article/10.3390/ijms26093952/s1.
