# Supplementary material for: Hallmarks of Cancer Cachexia: Sexual Dimorphism in Related Pathways
Source: Int J Mol Sci. 2025 Apr 22;26(9):3952. doi: 10.3390/ijms26093952 (PMC12071346; doi:10.3390/ijms26093952)
Supplement: Supplementary file 1 [file ijms-26-03952-s001.zip › ijms-3514138-supplementary.pdf]

Table S1. Supplementary data from the literature review that generated the graph in Figure 2.

Study	Age	Tumor type	Treatment	Total	Men (%)	Women (%)	Overall Prevalence of Cachexia (%)	Men Cachexia Prevalence (%)	Women Cachexia Prevalence (%)
**Study 1**	63.96 (±11.04)*	GI tract (65.3%), Lung (19.2%), Head/neck (11.4%), Breast (4.2%)	Different types of treatment	167	112 (67.1)	55 (32.9)	70.0	73.2	63.6
**Study 2**	67.4 (±11.6)*	GI- Colorectal, hepato-pancreato-biliary (83.3%); Non-GI - skin/soft tissue, breast and others (16.7%)	Pre-curative-intent cancer surgery	253	235 (92.9)	18 (7.1)	16.6	16.0	22.2
**Gastric, 2019**	66.49 (±10.8)*	Gastric cancer	Cancer surgery	575	433 (75.3)	124 (24.7)	48.0	35.3	42.7
**Head and neck cancer, 2019**	68 (60-75)**	Head and neck cancer	Chemoradiotherapy	44	42 (95.5)	2 (4.5)	11.4	12.0	0.0
**Oesophagus and gastric, 2021**	66 (59-71)**	Oesophagus, gastroesophageal junction, or stomach	Pretreatment	406	310 (76.3)	96 (23.7)	48.5	66.2	60.4
**Lung, 2022**	71 (47–86)**	Non-small-cell lung cancer - advanced (stage III or IV)	After definitive treatment	113	72 (63.7)	41 (36.3)	50.4	47.2	56.0
**Gastric 2, 2022**	66 (±14.0)*	Gastric cancer	Curative surgery	1215	886 (72.9)	329 (27.1)	26.5	23.7	34.0
**Colorectal, 2023**	79.6 (65-93)**	Colorectal cancer	Cancer surgery	64	28 (43.7)	36 (56.3)	36.0	25.0	44.4

*Presented as mean ± standard deviation. ** Presented as median [minimum; maximum].
